# CSF and Blood Levels of GFAP in Alexander Disease

**DOI:** 10.1523/ENEURO.0080-15.2015

**Published:** 2015-10-01

**Authors:** Paige L. Jany, Guillermo E. Agosta, William S. Benko, Jens C. Eickhoff, Stephanie R. Keller, Wolfgang Köehler, David Koeller, Soe Mar, Sakkubai Naidu, Jayne Marie Ness, Davide Pareyson, Deborah L. Renaud, Ettore Salsano, Raphael Schiffmann, Julie Simon, Adeline Vanderver, Florian Eichler, Marjo S. van der Knaap, Albee Messing

**Affiliations:** 1 Waisman Center and Department of Comparative Biosciences, University of Wisconsin-Madison, Madison, Wisconsin 53705; 2 Department of Child Neurology, Hospital Italiano School of Medicine, C1181ACH Buenos Aires, Argentina; 3 Developmental and Metabolic Neurology Branch, National Institute of Neurological Disorders and Stroke, National Institutes of Health, Bethesda, Maryland 20814; 4 Department of Biostatistics and Medical Informatics, University of Wisconsin-Madison, Madison, Wisconsin 53792; 5 Division of Pediatric Neurology, Emory University, Atlanta, Georgia 30322; 6 Chefarzt der Klinik für Neurologie und neurologische Intensivmedizin, D-04799 Wermsdorf, Germany; 7 Department of Molecular & Medical Genetics, Oregon Health & Science University, Portland, Oregon 97239; 8 Department of Pediatrics, Oregon Health & Science University, Portland, Oregon 97239; 9 Division of Pediatric Neurology, Washington University St. Louis, St. Louis, Missouri 63110; 10 Department of Neurology, Johns Hopkins University School of Medicine, Baltimore, Maryland 21205; 11 Division of Pediatric Neurology, University of Alabama-Birmingham, Birmingham, Alabama 35233; 12 Department of Clinical Neurosciences, IRCCS Foundation, C. Besta Neurological Institute, 20133 Milan, Italy; 13 Division of Child and Adolescent Neurology, Departments of Neurology and Pediatrics, Mayo Clinic, Rochester, Minnesota 55901; 14 Genomics Institute, Multi-Care Health System, Tacoma, Washington 98415; 15 Children's Research Institute, Children’s National Health System, Washington, DC 20010; 16 Massachusetts General Hospital, Boston, Massachusetts 02114; 17 Department of Child Neurology, Free University Medical Center, Amsterdam, 1007 MB, The Netherlands

**Keywords:** astrocyte, biomarker, GFAP

## Abstract

Alexander disease is a rare, progressive, and generally fatal neurological disorder that results from dominant mutations affecting the coding region of *GFAP*, the gene encoding glial fibrillary acidic protein, the major intermediate filament protein of astrocytes in the CNS. A key step in pathogenesis appears to be the accumulation of GFAP within astrocytes to excessive levels. Studies using mouse models indicate that the severity of the phenotype correlates with the level of expression, and suppression of GFAP expression and/or accumulation is one strategy that is being pursued as a potential treatment. With the goal of identifying biomarkers that indirectly reflect the levels of GFAP in brain parenchyma, we have assayed GFAP levels in two body fluids in humans that are readily accessible as biopsy sites: CSF and blood. We find that GFAP levels are consistently elevated in the CSF of patients with Alexander disease, but only occasionally and modestly elevated in blood. These results provide the foundation for future studies that will explore whether GFAP levels can serve as a convenient means to monitor the progression of disease and the response to treatment.

## Significance Statement

Glial fibrillary acidic protein (GFAP) is an intermediate filament protein that is predominantly expressed in astrocytes of the CNS. Although typically confined to the cytoplasm, GFAP is released at low levels into the extracellular space, and can appear at measurable levels in CSF and blood. Here we show that the fluid levels of GFAP increase markedly in individuals with Alexander disease, a genetic disorder that results from mutations in GFAP itself, particularly in the CSF. CSF analysis may therefore offer a relatively noninvasive means for indirectly monitoring the levels of GFAP in brain and assessing the efficacy of future experimental treatments that are designed to reduce these levels.

## Introduction

Alexander disease (AxD) is a progressive and generally fatal neurogenetic disorder, with ages of onset ranging from fetal through late adulthood, resulting from heterozygous dominant mutations in the astrocyte intermediate filament protein glial fibrillary acidic protein (GFAP; Brenner et al., 2001; Messing et al., 2012b). The hallmark feature of the pathology, cytoplasmic aggregates known as Rosenthal fibers within astrocytes, are composed of GFAP (Johnson and Bettica, 1989) along with a number of other proteins such as plectin (Tian et al., 2006), and the small stress proteins αB-crystallin and Hsp27 (Iwaki et al., 1989; Iwaki et al., 1993). Studies in mouse models have led to the conclusion that, unlike most of the other intermediate filament disorders, GFAP mutations act in a gain-of-function fashion, and that elevations of total GFAP levels may be a major factor in pathogenesis (Messing et al., 1998; Hagemann et al., 2006; Tanaka et al., 2007). Several strategies are now being pursued as potential treatments for AxD, chief among them being pharmacologic approaches for suppressing the expression of GFAP below its toxic threshold or interfering with other downstream effects of GFAP toxicity (Cho et al., 2010; Messing et al., 2010).

How one might assess the efficacy of any potential treatment is a major question. Although AxD is genetically homogeneous, the clinical presentations are quite varied, with age of onset ranging from fetal through late adulthood. The most widely used classification system is based simply on age of onset, and divides patients into early, juvenile, and adult categories (Russo et al., 1976). More recently, two different classification systems have been proposed that are more reflective of lesion location and symptoms (Prust et al., 2011; Yoshida et al., 2011). No consensus clinical scoring system exists for evaluating disease severity or monitoring progression, no clear genotype–phenotype relationships have been identified, and no prospective natural history studies have been performed. While existing MRI criteria are highly reliable as diagnostic tools (van der Knaap et al., 2001, 2005,2006), they are not suitable for quantifying disease severity or monitoring disease progression. Estimates of survival are also changing; Li et al. (2005), in an early review of 44 patients, calculated a median survival time from onset of 3.9 years for the early-onset group, while Prust et al. (2011), based on a much larger cohort of 215 patients, found a median 14-year survival time for the type I patients. Given the wide spectrum of clinical presentations and courses for AxD, there is a clear need to identify and evaluate biomarkers that can serve as surrogate indicators of the potential response to therapy.

One potential biomarker is GFAP itself. GFAP, despite being a cytoplasmic protein, is normally present at low levels in CSF and blood, and the levels in these fluids are increased in the context of a wide variety of injuries and diseases of the CNS (Liem and Messing, 2009; Petzold, 2015). Following traumatic brain injury, particularly ones that are acute and focal, serum GFAP levels rise, and serum GFAP level was recently adopted as a key biomarker for the TRACE-TBI study on common data elements (Yue et al., 2013). In one study of three AxD patients, Kyllerman et al. (2005) reported that CSF levels of GFAP were elevated in each one.

GFAP levels could conceivably serve as a biomarker in AxD in two distinct ways. First is the known association of GFAP release with injury or damage, as cited above; and, indeed, the lesions in AxD range from very subtle, without evident leukodystrophy, up to large cavitating lesions with loss of nearly all tissue elements (Messing and Goldman, 2004). Second is the proposed link between toxic accumulation of GFAP and pathogenesis—GFAP elevation is found in both human patient samples and mouse models (Hagemann et al., 2005, 2006; Tang et al., 2008; Walker et al., 2014), with the mice displaying a clear correlation between levels of GFAP expression and severity of phenotype (Messing et al., 1998; Hagemann et al., 2006). To determine whether GFAP levels can serve as a useful biomarker for AxD requires replication of the CSF findings from Kyllerman et al. (2005) in a larger cohort of patients, as well as measurement in more conveniently collected biopsy fluids such as blood.

## Materials and Methods

### AxD patient samples

The sole inclusion criterion for participation in this study was genetic confirmation of the diagnosis by sequencing of *GFAP*. Informed consent for studies of CSF was obtained following protocols approved by the institutional review boards (IRBs) at the University of Wisconsin-Madison, the Children’s National Medical Center, and the Mayo Clinic. Only leftover samples from a previous clinical use were permitted for study. Informed consent for studies of blood was obtained following protocols approved by IRBs at the University of Wisconsin-Madison, Children’s National Medical Center, Massachusetts General Hospital, Washington University in St. Louis, and the Mayo Clinic. Again, consents were obtained either through in-person interview or by telephone, with written confirmation. For samples collected in The Netherlands, Italy, and Germany, the principles outlined in the Declaration of Helsinki were followed.

### Control samples

Controls for CSF studies consisted of 24 deidentified samples collected by lumbar puncture (LP) for various purposes but considered within the range of normal for protein, glucose, and cell counts. The CSF controls were exempted from the requirement for consent. Additional information about the CSF control group (age, sex, and reason for LP) is given in **Table 1**. Controls for blood were obtained from apparently healthy adults of both sexes (27 males, 84 females), who were asked to exclude themselves if they had specific conditions (neurologic or psychiatric disorders, head or brain trauma within the past 12 months, type 1 diabetes, inflammatory bowel disease) or were taking specific medications within the past 3 months (clomipramine, amitriptyline, prednisone, dexamethasone, or tamoxifen). The exclusion criteria for plasma control samples were based on a literature review of conditions known or hypothesized to influence GFAP levels in CSF and blood (Liem and Messing, 2009) and a study specifically aimed at identifying pharmacological modifiers of GFAP expression (Cho et al., 2010).

**Table 1:** Control CSF samples (sorted by age at collection)

Age (years)	Sex	Reason for LP
0.08	M	Respiratory syncytial virus bronchiolitis
0.08	F	Acute respiratory failure
0.12	F	Acute life-threatening event
0.66	M	Vomiting
2.16	F	Gastrointestinal virus
2.25	M	Lymphoma
3.08	M	DiGeorge syndrome
3.75	F	Acute lymphocytic leukemia/chemotherapy evaluation
3.92	M	Acute lymphocytic leukemia/chemotherapy evaluation
4.08	M	Acute lymphocytic leukemia/chemotherapy evaluation
4.33	M	Acute lymphocytic leukemia/chemotherapy evaluation
5.25	M	Acute lymphocytic leukemia/chemotherapy evaluation
5.33	M	Acute lymphocytic leukemia
5.75	M	Acute lymphocytic leukemia/chemotherapy evaluation
6.92	F	Acute lymphocytic leukemia/chemotherapy evaluation
9.25	M	Acute lymphocytic leukemia/chemotherapy evaluation
9.58	F	Ehlers-Danlos syndrome, mental status change
12.33	M	Acute lymphocytic leukemia/chemotherapy evaluation
14.16	F	Acute lymphocytic leukemia/chemotherapy evaluation
14.75	F	Acute lymphocytic leukemia/chemotherapy evaluation
15.33	F	Idiopathic intracranial hypertension
16.66	M	Acute lymphocytic leukemia/chemotherapy evaluation
17.33	M	Acute lymphocytic leukemia/chemotherapy evaluation
19	M	Acute lymphocytic leukemia/chemotherapy evaluation

Deidentified control CSF samples are tabulated indicating sex and the clinical reason for LP. Age at collection is given in years. F, Female; M, male.

### Plasma preparation

Fresh samples of venous blood were collected into lavender-topped tubes that contained K_2_-EDTA as an anticoagulant to allow the preparation of plasma. The samples were centrifuged within 60 min of collection at 2500 relative centrifugal force for 15 min at room temperature, and the supernatant was immediately placed in a polypropylene tube and stored either on dry ice for shipping or in a −20°C freezer until shipping could be arranged. Upon arrival at the central laboratory, the samples were then thawed, divided into aliquots, and stored at −80°C until further analysis. Three blood samples were collected as serum rather than plasma, and so were considered nonstandard. Statistical analyses were repeated with or without these samples, and the results were the same.

### Quantitation of GFAP

GFAP levels in CSF and blood were quantitated using a sandwich ELISA, as previously described (Jany et al., 2013). The capture antibodies consisted of a cocktail of monoclonal antibodies (SMI-26R, Covance) diluted in PBS (catalog #BP3994, Fisher). Plates were blocked with 5% milk in PBS before the addition of samples or standards diluted in PBS with 0.05% Tween 20 and 1% BSA (catalog #A7030, Sigma-Aldrich), with each sample analyzed in triplicate. Antibody incubation steps were performed in 5% milk-PBS, and washing steps were performed in PBS-Tween 20. Standard curves were generated using bovine GFAP (catalog #RDI-PRO62007, Fitzgerald Industries International), and reaction volumes consisted of 100 μl/well. CSF and plasma samples were initially diluted 1:1 with ELISA buffer, though in some cases higher dilutions were necessary to bring the values within the linear range of the assay. GFAP values were expressed as ng/L. Under these conditions, the lower limit of detection was 11 ng/L, and the biological limit of detection (BLD; after accounting for the minimum 1:1 dilution with reaction buffer) was 46 ng/L. The intra-assay coefficient of variation, determined using the bovine GFAP standard at 33 ng/L in 10 sets of triplicate wells, was 13%. The interassay coefficient of variation, determined using pooled CSF samples taken from *GFAP^Tg^* mice that overexpress wild-type human GFAP, was 11%. CSF and plasma samples from *Gfap*-null mice gave readings that were below the BLD in this assay (data not shown), thus validating its specificity. In addition, plasma samples from *Gfap*-null mice were spiked with known concentrations of purified bovine GFAP to verify that the 1:1 dilutions of plasma used here did not interfere with the sensitivity of the assay (data not shown). All animal procedures were approved by the Animal Care and Use Committee for the Graduate School at the University of Wisconsin-Madison.

### Statistics

GFAP levels obtained from CSF and blood samples were summarized in terms of medians and ranges. To determine a reference range for GFAP levels in blood samples from healthy control samples, we took into account the BLD of the assay (46 ng/L). Samples yielding values lower than this limit were treated as censored values at 46 ng/L in the analysis. Due to the presence of censoring, semiparametric quantile regression was conducted (Richardson and Ciampi, 2003). Overall, the quantile regression analysis indicated that there was no age or gender effect on the GFAP values in the control subjects. The nonparametric Wilcoxon rank sum test was used to compare GFAP levels between AxD case patients and control subjects. Analogously, within AxD case patients, the nonparametric Wilcoxon rank test was used to conduct comparisons among subgroups. The Sidak correction was applied when performing multiple comparisons. Ninety-five percent confidence intervals (CIs) for the differences in medians between AxD case patients and control subjects were constructed using the nonparametric bootstrap method. All *p* values were two sided, and *p* < 0.5 was used to determine statistical significance. Statistical analyses were conducted using SAS version 9.3 software (SAS Institute).

## Results

### Patient population

Samples were collected from AxD patients with confirmed mutations in *GFAP*. Those patients for whom leftover clinical CSF samples were available included five females and seven males, ranging in age from 3.7 to 46 years. Those patients from whom blood samples were collected included 26 females and 22 males, ranging in age from <1 to 65 years of age. Both blood and CSF samples were available for 10 of these patients. Information for each patient regarding gender, specific mutation, age of first symptom, age at collection of samples, and age at death (if relevant) is provided in **Table 2**.

**Table 2:** AxD patient samples

**Patient no.**	**Mutation**	**Sex**	**Age at illness onset (years)**	**Age at sample collection (years)**		**Death**	**Reference**
				**CSF**	**Blood**		
1	R70Q	F	40.5		44.67		Patient 12 in Caroli et al. (2007)
2	N77S	M	0.16		1.75		
3	N77S	F	1		3.26		
4	N77S, S152L	F	0.58		19.19		
5	R79C	M	0.5	17.28	20.84		Patient 6 in Li et al. (2005)
6	R79C	M	0.25		6.20		Matarese and Renaud (2008)
7	R79C	F	4*	4*			
8	R79C	F	0.5		2.02		
9	R79G	F	0.29		2.25	2.65	
10	R79H	F	0.58	6.27	7.11		
11	R79H	F	0.5		1.92		
12	R79H	F	1.25		10.51		
13	R79H	F	0		3.07		
14	R79L	M	0.5	4.65	4.65		
15	R88C	M	4		13.36		Patient 10 in Gorospe et al. (2002)
16	R88C	M	0.75		2.24		
17	R88C	M	2		5.77		
18	R88C	M	10	15.78	17.87		
19	R88C†	F	10		40.95		Patient 3 in van der Knaap et al. (2006)
20	R88C†	M	10	17.32	21.62		Patient 4 in van der Knaap et al. (2006)
21	R105W‡	F	6		15.74		
22	L123P	M	50		56.72		
23	E207Q	M	10.5		22.11	22.52	Patient 12 in Li et al. (2005) Patient 7 in van der Knaap et al. (2005)
24	L231H†	M	50		64.88		Delnooz et al. (2008)
25	L231H†	M	NA		34.85		
26	R239C	M	0.5		2.50		
27	R239C	F	1.5		2.10		
28	R239C	F	7.00		9.20		
29	R239C	F	1.67		1.90		
30	R239H	F	0.29		0.72	1.14	
31	R239H	F	0		0.96	1.00	
32	R239P	M	2	23.85	23.85		Patient 1 in van der Knaap et al. (2005)
33	R239P	M	1.5		3.25		
34	S247P	M	10		36.68		Patient A.II.d in Messing et al. (2012a)
35	R258P	M	0	3.72	2.61		
36	R270-A272del	F	<0.25		3.93		
37	Q290E	F	12		13.82		Patient 1 in Barreau et al. (2011)
38	E362Q	F	5		20.52		
39	E371Q	M	<1		9.78		
40	E373A	F	34		36.60		
41	E374G	F	0	1.91		12.82	Patient 40 in Li et al. (2005)
42	S398F	F	45	45.59	45.84		
43	S398Y	F	51*		56.67		Patient 9 in Pareyson et al. (2008) and Farina et al. (2008)
44	M415I†	F	40		52.22		de Souza Rezende et al. (2012)
45	M415I†, D157N§	F	4		19.46		de Souza Rezende et al. (2012)
46	R416W	M	14	31.47	31.19	33.63	
47	R416W	M	13		31.68		Patient 3 in Pareyson et al. (2008) and Farina et al. (2008)
48	R416W	M	6		8.14		
49	R416W	F	16		25.93		
50	Q426L	F	30	44.15	44.15		Patient C.II.1 in Messing et al. (2012a)

Information regarding each patient who contributed blood and/or CSF samples is shown, including gender, GFAP mutation, age of illness onset, age at sample collection, and age at death (if relevant), and sorted by GFAP mutation. For some patients, the age of illness onset was estimated (*) or the patient was asymptomatic but had a familial history of AxD (NA). All ages are given in years. References to prior publications containing additional clinical details about particular patients are also given, if available. F, Female; M, male.

* Age of onset was estimated.

† Parent–child duos are shown together on consecutive lines (19-20, 24-25, and 44-45).

‡ The pathogenicity of the R105 mutation is uncertain.

§ The D157N mutation is considered a benign variant, but its impact in a compound heterozygote is not known.

### GFAP levels in CSF

**Figure 1.**
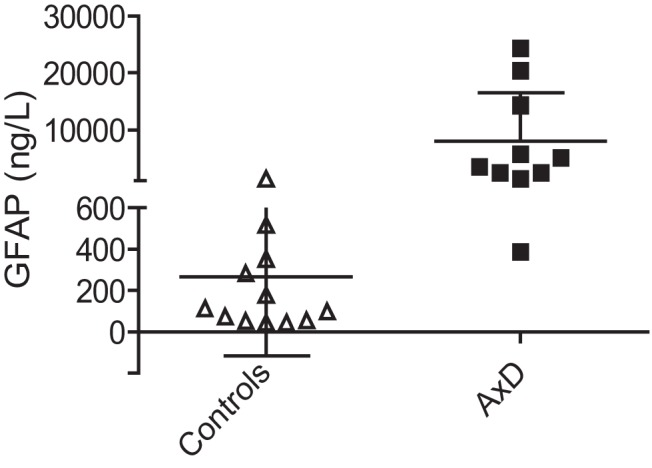
GFAP levels in CSF. GFAP levels (in ng/L) in CSF of AxD patients and control subjects are shown; data are presented as the mean ± 1 SD on a split linear scale for the *y*-axis. GFAP levels in samples from AxD patients are significantly elevated compared with those from control subjects. Each data point represents one individual (AxD patients: *n* = 6 males, 4 females; control subjects, *n* = 7 males, 5 females; Wilcoxon rank sum test, *p* < 0.001^a^).

We established a reference range for CSF controls in our assay using a set of 24 samples, and found the mean GFAP level to be 249 ng/L (median, 133 ng/L; range, 46-1386 ng/L, with one value falling below the BLD). The highest values in the control group came from one child with lymphoma (site unspecified) and one child with an “acute life-threatening event” (type unspecified). CSF samples were initially available for 10 AxD patients, and in a single assay all of the AxD patient samples were run alongside 12 samples from the control subjects (the control subjects chosen to span the full range as previously identified; **Fig. 1**). Considered as a group, GFAP levels in the AxD patients were significantly elevated compared with the control subjects (patients: median, 4292 ng/L; range, 387-24272 ng/L; control subjects: median, 103 ng/L; range, 46-1386 ng/L; *p* < 0.001^a^). Considered as individuals, GFAP levels in 9 of the 10 samples from AxD patients were elevated compared with those from the control population. Because of the paucity of samples, this experiment was replicated once, with similar results. Two additional samples were subsequently received (from patients 35 and 50) and analyzed separately, and yielded values of 2721 and 1749 ng/L, respectively. Individual GFAP values for each AxD patient in relation to age, gender, genotype, and duration of illness are shown in **Table 3**.

**Table 3:** GFAP levels in CSF and blood of AxD patients

**Patient no.**	**Mutation**	**Sex**	**GFAP levels** **(ng/L)**		**Age at illness onset** **(years)**	**Duration of illness** **(years)**	
			**CSF**	**Blood**		**CSF**	**Blood**
1	R70Q	F		64	40.5		4.17
2	N77S	M		1640	0.16		1.59
3	N77S	F		802	1		2.26
4	N77S, S152L	F		480	0.58		18.61
5	R79C	M	5803	256	0.5	16.78	20.34
6	R79C	M		1864	0.25		5.95
7	R79C	F	3489		3.9*	0.10*	
8	R79C	F		426	0.5		1.52
9	R79G	F		1201	0.29		1.96
10	R79H	F	14290	1154	0.58	5.69	6.53
11	R79H	F		255	0.5		1.42
12	R79H	F		302	1.25		9.26
13	R79H	F		572	0		3.07
14	R79L	M	20355	1925	0.5	4.15	4.15
15	R88C	M		238	4		9.36
16	R88C	M		46	0.75		1.49
17	R88C	M		329	2		3.77
18	R88C	M	5095	132	10	5.78	7.87
19	R88C†	F		1068	10		30.95
20	R88C	M	2493	122	10	7.32	11.62
21	R105W‡	F		105	6		9.74
22	L123P	M		219	50		6.72
23	E207Q	M		751	10.5		11.61
24	L231H†	M		95	50		14.88
25	L231H	M		243	N/A		N/A
26	R239C	M		1007	0.5		2.00
27	R239C	F		791	1.5		0.60
28	R239C	F		504	7		2.20
29	R239C	F		237	1.67		0.23
30	R239H	F		169	0.29		0.43
31	R239H	F		711	0		0.96
32	R239P	M	24272	713	2	21.85	21.85
33	R239P	M		461	1.5		1.75
34	S247P	M		248	10		26.68
35	R258P	M	2721	750	0		2.61
36	R270-A272del	F		1314	0.25*		3.68*
37	Q290E	F		446	12		1.82
38	E362Q	F		322	5		15.52
39	E371Q	M		704	0.9*		8.88*
40	E373A	F		187	34		2.60
41	E374G	F	387		0	1.91	
42	S398F	F	1402	505	45	0.59	0.84
43	S398Y	F		112	51*		5.67*
44	M415I†	F		46	40		12.22
45	M415I, D157N§	F		571	4		15.46
46	R416W	M	2478	62	14	17.47	17.19
47	R416W	M		545	13		18.68
48	R416W	M		712	6		2.14
49	R416W	F		64	16		9.93
50	Q426L	F	1749	114	30	14.15	14.15

GFAP concentrations (in ng/L) in CSF and blood of individual AxD patients. Patient 25 was asymptomatic at the time of collection. Duration of illness is defined as the age at sample collection less the age at illness onset, using the values shown in Table 2. Parent–child duos are shown together (19-20, 24-25, and 44-45), as in Table 2. All blood samples are plasma, except for three (9, 16, and 50), which are serum. N/A, not applicable.

* Age at illness onset and duration of illness were estimated.

† Parent–child duos are shown together on consecutive lines (19-20, 24-25, and 44-45).

‡ The pathogenicity of the R105 mutation is uncertain.

§ The D157N mutation is considered a benign variant, but its impact in a compound heterozygote is not known.

### GFAP levels in blood

**Figure 2.**
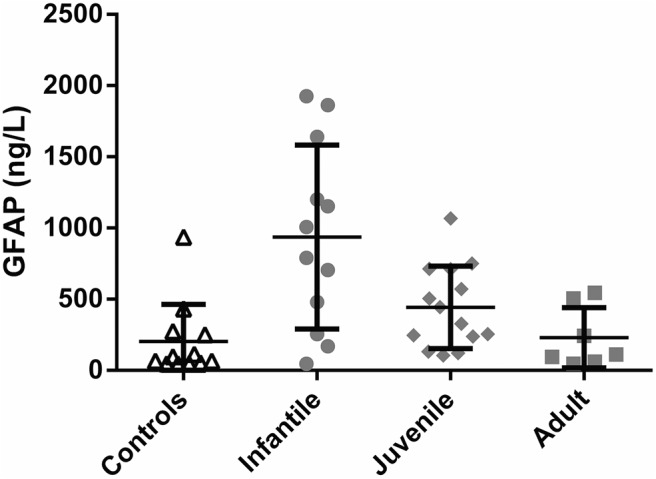
GFAP levels in blood. GFAP levels (in ng/L) in blood of control subjects compared with AxD patients grouped by age of onset (infantile onset, 0-2; juvenile onset, >2-13; adult onset, >13). Each data point represents one individual. Horizontal bars indicate the mean, and the error bars indicate ±1 SD. The infantile group (Wilcoxcon rank sum test, *p* = 0.002^b^) and the juvenile group (Wilcoxon rank sum test, *p* = 0.025^c^) are significantly different than control subjects.

We established a reference range for plasma controls in our assay using a set of 111 samples obtained from healthy volunteers. Run in their entirety in a single assay, 65 of these samples yielded values in the detectable range, with a median of 61 ng/L and a 95% CI of 46-861 ng/L. This experiment was replicated three times on subsets of control subjects with similar results. Plasma samples from 33 AxD patients were then run in a single assay alongside a subset of samples from 12 control subjects. The GFAP concentrations in the subset of samples from control subjects compared with the full set of 111 plasma samples from control subjects revealed no significant differences (data not shown). Considered as a group, GFAP levels in the AxD patients were significantly elevated in the AxD case patients with infantile (*p* = 0.002^b^) and juvenile (*p* = 0.025^c^) onsets compared with those in the control subjects (**Fig. 2**). This assay was repeated three times with similar results. Individual GFAP values for each AxD patient are shown in **Table 3**.

Subsequent to the experiments described above on the large set of AxD patients, 12 additional samples were received, which were analyzed on three separate occasions (with smaller numbers of control subjects to verify the consistency of the assay). In these latter patients (**Tables 2, 3**, patients 1, 8, 13, 22, 29, 31, 35-36, 38, 40, 49-50), plasma values ranged from 64 to 1314 ng/L, only patient 36 being higher than the 95% CI established for control subjects.

**Figure 3.**
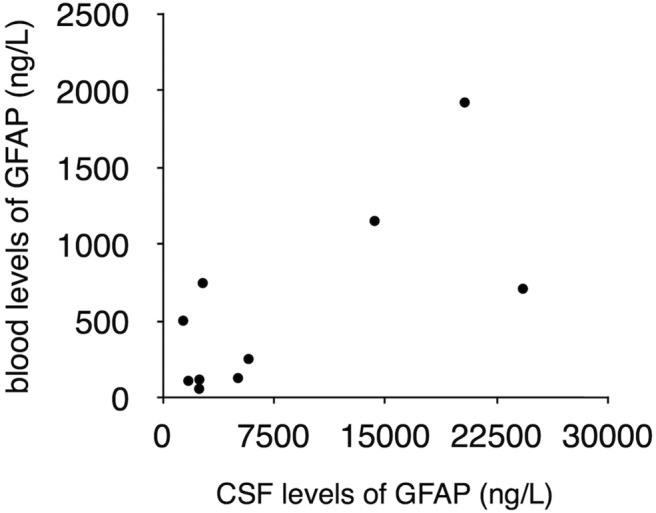
Within-subject comparison of GFAP levels in CSF and blood. GFAP levels in blood are shown as a function of the levels in CSF for the ten patients for whom both types of samples were available. CSF values were consistently higher than blood values. However, as indicated by the ages at collection as given in Table 1, the samples were not contemporaneous. No statistical analysis was performed due to the low number of samples.

Both CSF and blood samples were available for 10 AxD patients (though usually collected at different ages; **Table 3**). For each of these patients, CSF values were significantly higher than the blood values. Although the number of patients was too low for valid statistical analysis, it appears that the blood levels may only exceed the 95% CIs of control subjects when the CSF values are above a certain threshold (**Fig. 3**).

### GFAP levels in relation to clinical features

We analyzed the individual blood and CSF values of GFAP in relation to several aspects of the clinical and genetic histories. Additional clinical information about each AxD patient is given in **Table 4**. With respect to specific genotype, the 12 CSF samples came from patients with 10 different mutations, and only 2 each for the R79C and R88C mutations, so no conclusions were possible. For blood samples, five mutations were represented by four to six patients (R79C, R79H, R88C, R239C, and R416W), and two samples were available for the one genotype that is generally considered severe (R239H); but the values for GFAP were all highly variable in these groups. Hence, there does not appear to be any direct connection between genotype and the levels of GFAP detected in these assays. In addition, when gender was considered as a variable, there was no significant difference between males and females.

**Table 4:** Clinical information on AxD patients

**Patient no.**	**Mutation**	**Sex**	**Onset**	**First symptom**	**Highest cognitive**	**Highest motor**	**Main deterioration**	**Age at loss of unassisted walking**
1	R70Q	F	40.5	Ataxia	Normal	Walks without support	Gait	No
2	N77S	M	0.16	Frequent arching, seizures	Severe ID	None at all	Severe spasticity, intractable seizures	N/A
3	N77S	F	1	Speech and motor delay	Moderate ID	Walks without support, but wide-based gait	ND	ND
4	N77S, S152L	F	0.58	Seizures	Normal	Walks with support	Motor and language deterioration	Yet to walk without support
5	R79C	M	0.5	Motor delay	Mild ID	Walks without support	Spastic tetraparesis, cognitive problems	14 years
6	R79C	M	0.25	Macrocephaly, developmental delay	Normal	Walks without support	None	No
7	R79C	F	4*	ND	ND	ND	ND	ND
8	R79C	F	0.5	Developmental delays in speech, walking, hypotonia	Mild–moderate ID	Standing	Facial droop after concussion	Yet to walk without support
9	R79G	F	0.29	Seizures	Severe ID	Sitting without support	Loss of sitting	Never walked without support
10	R79H	F	0.58	Seizure	Mild ID	Walks without support	Ataxia	ND
11	R79H	F	0.5	Arching back and eye rolling upward	Normal	Walks with support	Seizures	Yet to walk without support
12	R79H	F	1.25	Seizure	Moderate ID	Walks without support	Motor skills, cognition	No
13	R79H	F	0	Hypotonia	Severe ID	Reaching for objects	N/A	N/A
14	R79L	M	0.5	Progressive macrocephaly; slowed development	Moderate ID	Walks a few steps without support	None	No
15	R88C	M	4	Short stature, followed by slowed cognitive development	Mild ID	Walks without support	Progressive dysarthria, cognitive delay	No
16	R88C	M	0.75	Macrocephaly, developmental delay	Mild ID	Walks with support	None	Yet to walk without support
17	R88C	M	2	Seizure	Mild ID	Walks without support	Motor decline, seizures, bulbar problems	5 years
18	R88C	M	10	Deterioration in academic skills	Mild ID	Walks without support	Neurocognitive decline and spasticity	No
19	R88C†	F	10	Vomiting, anorexia	Normal	Walks without support	Scoliosis, gait	30 years
								
20	R88C†	M	10	Incoordination	Normal	Walks without support	Scoliosis, gait, some cognitive decline	No
21	R105W‡	F	6	Memory, math and spelling, behavior	Mild ID	Walks without support	None	No
22	L123P	M	50	Progressive gait problems, inbalance	Normal	Walks without support	Bulbar dysfunction	ND
23	E207Q	M	10.5	Scoliosis, followed by abnormal gait, fatigue, and weakness	Normal	Walks without support	Difficulty walking, urinary incontinence	22 years
24	L231H†	M	50	Ataxia	Normal	Walks without support	Ataxia	63 years
25	L231H†	M	na	None	Normal	Walks without support	None	No
26	R239C	M	0.5	Macrocephaly, developmental delay	Severe ID	Standing with support	Swallowing, tone, hydrocephalus	Yet to walk without support
27	R239C	F	1.5	Hypotonia, gross motor delay, macrocephaly	Severe ID	Walks with support	Failure to thrive, emesis, but no regression	Yet to walk without support
28	R239C	F	7	Choking episodes	Mild ID	Walks without support	Gait deterioration, dysarthria, urinary incontinence	No
29	R239C	F	1.67	Intermittent ataxia	Normal	Walks without support	Occasional unsteadiness	No
30	R239H	F	0.29	Vomiting, hypotonia, minimal development	Only social contact	None at all	Progressive bulbar dysfunction	Never walked without support
31	R239H	F	0	Hydrocephalus, minimal development	Severe ID	None	N/A	N/A
32	R239P	M	2	Vomiting, deterioration of gait	Moderate ID	Walks without support	Mild deterioration of gait	No
33	R239P	M	1.5	Speech and motor delay	Mild ID	Walks with support	None	Yet to walk without support
34	S247P	M	10	Severe morning emesis	Normal	Walks without support	Sleep apnea	No
35	R258P	M	0	Macrocephaly, hypotonia	Mild–moderate ID	Walks without support	Seizures, dysarthria, ataxia	No
36	R270-A272del	F	<0.25	Motor delay, macrocephaly	Severe ID	Very limited	N/A	N/A
37	Q290E	F	12	Worsening migraines	Normal	Walks without support	No	No
38	E362Q	F	5	Seizures, ataxia, rigidity	Normal	Normal	Dysarthria, short-term memory, executive function	No
39	E371Q	M	<1	Motor delay	Mild ID	Walks without support	Neurocognitive delay	No
40	E373A	F	34	Numbness, burning sensation	Normal	Normal gait	Fatigue, balance, bladder	No
41	E374G	F	0	Hypotonia, lack of development	Moderate ID	Walks with support	Lost all skills, frequent vomiting	Never walked without support
42	S398F	F	45	Dysarthria	Normal	Walks without support	Ataxia, palatal tremor	No
43	S398Y	F	51*	MRI after subarachnoid hemorrhage at 51 years, mild urinary urgency at 56 years	Normal	Walks without support	Urinary urge-incontinence, unsteadiness	No
44	M415I†	F	40	Balance difficulties	Normal	Walks without support	Speech, urinary, headache	No
45	M415I†, D157N§	F	4	Ataxia	Normal	Walks without support	Urinary retention, bulbar dysfunction	∼8 years
46	R416W	M	14	Behavior and gait problems; single seizure	Low normal	Walks without support	Ataxia, dysarthria, behavior	18 years
47	R416W	M	13	Dysarthria, dysphagia	Normal	Walks without support	Cognitive impairment, neurogenic bladder, obstructive sleep apnea, palatal tremor	29 years
48	R416W	M	6	Febrile seizure	Low normal	Walks without support	Mild proximal weakness	No
49	R416W	F	16	Balance, bladder	Normal	Walks without support	Balance coordination, weakness, swallowing, hallucinations	No
50	Q426L	F	30	Urinary incontinence, neurogenic bladder	Normal	Normal	Exercise intolerance	45

Information regarding each patient is shown including age of onset, nature of first symptom, highest cognitive level, highest motor level, major deterioration (if any), and age at loss of unassisted walking (if it occurred). All ages are given in years. ID, Intellectual disability; N/A, not applicable; ND, not determined or unknown; F, female; M, male.

* Age of onset was estimated.

† Parent-child duos are shown together on consecutive lines (19-20, 24-25, and 44-45).

‡ The pathogenicity of the R105 mutation is uncertain.

§ The D157N mutation is considered a benign variant, but its impact in a compound heterozygote is not known.

The number of CSF samples was so small that we could not establish any particular connection to age of onset of illness, age at sample collection, or duration of illness (i.e., the difference between the two time points). For blood, the GFAP values were significantly correlated with the age of illness onset and age at sample collection (which is likely linked to the age of onset), but not duration of illness. Four blood samples were from patients sampled within months of their deaths (patients 9, 23, 30, and 31), but only one of these had a value that was above the 95% CI. If the data are analyzed according to the clinical classification scheme of Russo et al. (1976), which is explicitly tied to age of illness onset, then the GFAP values in patient CSF samples were significantly higher than those in control subjects in all three groups (infantile, juvenile, and adult onset of illness), whereas the values in blood were higher in just the infantile and juvenile groups but not in the adult group. The recent classification scheme of Prust et al. (2011) relies more on clinical features (and hence anatomy) rather than age of illness onset, but still shows a marked age difference. While we did not have sufficient information to allow precise classification according to the criteria of Prust et al. (2011), if one uses an age of AxD onset of <4 or >4 year as a surrogate for classifying patients as either type I or type II, respectively, then it is clear that the CSF values for AxD patients are significantly elevated in both groups, whereas the blood values are significantly elevated only in the putative type I patients (type I patients vs control subjects, *p* = 0.001^d^; type II patients vs control subjects, *p* = 0.203^e^). Additional information about the statistical tests used throughout and the confidence intervals for each set of comparisons is given in **Table 5**.

**Table 5:** Statistical table

	Data structure	Type of test	95% CI
a	Quantitative scale, non-normally distributed	Wilcoxon rank sum test	1876–14226 ng/L
b	Quantitative scale, non-normally distributed	Wilcoxon rank sum test	191–1015 ng/L
c	Quantitative scale, non-normally distributed	Wilcoxon rank sum test	40–563 ng/L
d	Quantitative scale, non-normally distributed	Wilcoxon rank sum test	250–710 ng/L
e	Quantitative scale, non-normally distributed	Wilcoxon rank sum test	−110–381 ng/L

## Discussion

In an earlier study of three AxD patients, Kyllerman et al. (2005) found that GFAP levels in CSF were elevated compared with a previously analyzed reference range of control subjects. We here confirm the consistent elevation of GFAP levels in CSF samples in a larger cohort of patients, analyzed at the same time as control subjects. We further report that GFAP levels are also elevated above the control range in blood samples, but only in a subset of patients. Our results provide the necessary foundation for future studies aimed at developing appropriate biomarkers of disease severity, progression, and response to treatments.

Why GFAP levels in CSF (and occasionally blood) rise in AxD patients is not clear. Previous work in mouse models suggests a linear relation between the levels in parenchyma (presumably largely intracellular) and that in the fluids (Jany et al., 2013), and it is known that GFAP levels are elevated in brain lysates from AxD patients (Tang et al., 2008; Walker et al., 2014). That the infantile- and juvenile-onset groups display higher fluid levels than adult-onset patients may reflect underlying differences in their parenchymal levels, but such direct correlations remain to be established. The precise origin and form of extracellular GFAP are also not certain, but presumably result from either astrocyte death or sublethal injury that increases the permeability of the plasma membrane (Liem and Messing, 2009; Petzold, 2015). GFAP is not known to be secreted, as has been reported for vimentin from macrophages (Mor-Vaknin et al., 2003), though unpublished studies suggest that it might be exported via exosomes (K. Glebov, personal communication). Recent studies of the newly discovered “glymphatic” pathway for solute flow in the brain suggest that GFAP may reach the blood through paravascular channels and olfactory pathways rather than a leaky blood–brain barrier (Plog et al., 2015). However, it is possible that glymphatic clearance itself, which is dependent on the astrocytic water channel AQP4, is also affected in AxD patients.

Individuals with AxD experience several associated conditions that plausibly could influence GFAP expression and levels in biofluids. Seizures are seen in most infantile or type I AxD patients (Brenner et al., 2009; Prust et al., 2011), and kindling has been shown in rat models to transiently increase the expression of GFAP even in the contralateral hippocampus (Steward et al., 1991). Nevertheless, in our sample we found no obvious connection linking GFAP levels with the co-occurrence or degree of control of seizures. Conditions involving more obvious destruction of brain parenchyma, such as hydrocephalus (Tullberg et al., 1998; Beems et al., 2003; Petzold et al., 2004) or cavitating lesions (Rosengren et al., 1994), might also be expected to contribute to increased spillage of GFAP into the extracellular space, and subsequently into CSF and blood. Nevertheless, GFAP blood levels were elevated in only one of the five patients noted to have hydrocephalus. Whether certain features of MRI findings in individual AxD patients, such as cavitation or simple contrast enhancement (reflecting breakdown of the blood–brain barrier), predict changes in GFAP levels in CSF and blood is a topic for future study.

We recognize a number of limitations to our study. First is the problem of age matching in our samples, particularly with respect to blood. Our IRB protocol limited the collection of blood from healthy control subjects to those >18 years of age, whereas 27 of our 48 AxD patient samples were below this age. However, previous studies of GFAP in CSF and blood found no relation to age or sex (Petzold et al., 2004; Mayer et al., 2013), though with a small sample Rosengren et al. (1992) found that adult CSF values were slightly higher than those in children. Second, while the processing of the blood samples in our study was well defined and standardized, for CSF we could access only leftover clinical samples, which may have varied considerably with respect to the interval between collection and freezing. Our assays did not distinguish between full-length protein and forms that are truncated through degradation, but previous studies (Missler et al., 1999; Petzold et al., 2004) have shown that GFAP is stable for several days at 4°C and is not affected by multiple freeze–thaw cycles. In addition, circadian rhythms influence the production rate of CSF (Nilsson et al., 1992), and the levels of at least some biomarkers (e.g., amyloid β) are known to vary depending on the time of collection (Bateman et al., 2007). We note that the quantitation of proteins involved in aggregation disorders may be compromised by the “hook” effect, but the impact would be to underestimate the degree of elevation, particularly when assaying tissues where the aggregates typically reside (Lu et al., 2011; Petzold, 2015). Finally, we have examined only samples taken at one time point during the disease process; for many of the AxD patients who contributed CSF samples, they were taken at different times than the blood samples. Hence, our data cannot be used to establish direct connections between the values in blood and CSF, nor can we reach any conclusions about how GFAP levels might change in an individual during progression of disease.

Ultimately, a panel of biomarkers may prove more informative than focusing on just one. Several candidates for consideration in such a panel could include markers of neuronal or oligodendroglial injury (e.g., neuron-specific enolase, UCH-L1, neurofilament-H, MBP). A recent proteomic study of CSF in one mouse model of AxD (Cunningham et al., 2013) suggested additional potential biomarkers that may be of interest, including cathepsins and creatine kinase M.

## Synthesis

The decision was a result of the Reviewing Editor Robert Kalb and the peer reviewers coming together and discussing their recommendations until a consensus was reached. A fact-based synthesis statement explaining their decision and outlining what is needed to prepare a revision is listed below. The following reviewers agreed to reveal their identity: Gihan Tennekoon, James Goldman

Both reviewers found significant merit to the work. Relatively small issues were raised including tightness of language in the Introduction and Discussion and replicates/COV. Once these issues are addressed, the ms. would be appropriate for publication in eNeuron
